# Gender and Age Differentials in Prevalence and Pattern of Nine Chronic Diseases Among Older Adults in India: An Analysis Based on Longitudinal Ageing Study in India

**DOI:** 10.1111/jch.70069

**Published:** 2025-05-10

**Authors:** Mrigesh Bhatia, Laxmi Kant Dwivedi, Priti Maurya, Sameer Dawoodi, Waquar Ahmed, Somnath Jana, Priyanka Dixit

**Affiliations:** ^1^ Department of Health Policy London School of Economics London UK; ^2^ Department of Survey Research & Data Analytics International Institute for Population Sciences Mumbai India; ^3^ International Institute for Population Sciences Mumbai India; ^4^ Gastroenterology & Hepatology SUNY Downstate Health Sciences University Brooklyn New York USA; ^5^ School of Health Systems Studies Tata Institute of Social Sciences (TISS) Mumbai India; ^6^ International Institute for Population Sciences Mumbai India; ^7^ School of Health Systems Studies Tata Institute of Social Sciences (TISS) Mumbai India

**Keywords:** Bone and joint Diseases, Cancer, Chronic Heart Diseases, chronic diseases, Chronic lung Diseases, Diabetes, gender disparities, Hypertension, High Cholesterol, Longitudinal Ageing Study in India (LASI), older adults, Psychiatric Diseases, Stroke

## Abstract

Non‐communicable diseases (NCDs) account for a major portion of morbidity and mortality worldwide, with older persons being especially vulnerable due to age‐related health concerns. The burden of chronic diseases among India's aging population is understudied, particularly in terms of gender and age differences. The study utilized data from the Longitudinal Ageing Study in India (LASI), wave 1 (2017–18), comprising a sample of 66,606 older adults aged 45 years and above. We performed a Mmultivariable logistic regression analysis to examine the age‐gender differences in the prevalence and patterns of nine chronic diseases, namely hypertension, diabetes, cancer, chronic heart disease, stroke, psychiatric disorders, chronic lung disease, bone and joint diseases, and high cholesterol among older adults after adjusting for various socio‐demographic and lifestyle factors. Compared to male respondents, female older respondents were less likely to have diabetes and stroke (in the 45–59 and 60–69 years age groups), chronic lung diseases (in the 45–59 years age group), and chronic heart diseases (in the 60–69 years age group). Conversely, in the 70 years and above age group, older female respondents had higher odds of having hypertension and bone and joint diseases compared to male respondents. The current study revealed significant gender and age‐related differences in the prevalence and odds of all the nine selected diseases even adjusted for potential confounding factors. The findings highlight how urgently age‐ and gender‐specific treatments are needed to reduce inequities in chronic diseases, boost positive health outcomes, and improve the quality of life for India's aging populations.

## 1 Introduction

Non‐communicable diseases (NCDs) represented the largest global burden and were the only disease group to show an increase in disability‐adjusted life years (DALYs) between 2010 and 2021, rising from 1.47 billion in 2010 to 1.73 billion in 2021 [1]. This increase was primarily attributed to aging and population growth, as 165 of 204 countries experienced population growth during this period. Age‐standardized DALY rates for NCDs, however, decreased by 6.4% during this same period [1]. NCDs also accounted for 74.36% of all‐cause mortality worldwide in 2019 [2]. NCDs affect individuals across all age groups, regions, and countries. While these conditions are commonly linked to older populations, evidence indicates that 17 million NCD‐related deaths occur before the age of 70. Notably, an estimated 86% of these premature deaths take lace in low‐ and middle‐income countries [3].

The relationship between aging and chronic diseases is multifaceted, with age being a primary risk factor for the onset of multiple chronic conditions, often leading to multimorbidity [4]. However, the burden of chronic diseases is not uniformly distributed across different age groups as significant gender and age differentials also exist, influencing health outcomes and disease patterns among older adults [5, 6, 7]. A prior studies proposed that the variations in morbidity between men and women may be more attributable to varying levels of exposure to risk factors rather than differences in vulnerability [8]. Exposure to specific risk factors, such as being overweight in the case of arthritis or smoking in the case of lung diseases, has been identified as a contributing factor to the gender disparities observed in certain morbidities [9]. Chronic illnesses can lead to functional disabilities, limiting the ability of older adults to engage in daily activities and social interactions [10]. This functional decline is often exacerbated by socioeconomic factors, which can further influence health disparities among older populations [5].

The disparities in health outcomes and chronic morbidity between men and women are thought to necessitate multifactorial explanations, as they arise from the combined effects of social determinants of health alongside genetic and biological differences [12]. Several studies in the literature have demonstrated that men and women are exposed to these determinants in different ways and that the pathways leading to structural, behavioral and psychosocial factors influencing health and morbidity differ by gender. s Women exhibit distinct responses to these factors compared to men, reflecting a gender‐specific response [11, 12, 13, 14, 15, 16]. Men tend to experience a higher incidence of more fatal diseases, such as heart disease and cardiovascular problems [9, 17, 18].

A recent study reported that the age‐adjusted prevalence of heart disease, coronary heart disease (CHD), hypertension and stroke were 12.6%, 7.4%, 26.1%, and 3.1%, respectively among men; however in females, the prevalence rates were 10.1% for heart disease, 4.1% for CHD, 23.5% for hypertension, and 2.6% for stroke [19]. The reduction in life expectancy associated with diabetes decreases as the age at diagnosis increases. However, due to the growing prevalence of diabetes, the total number of years lived with the condition has risen significantly, with an increase of 156% for men and 70% for women overall [20].

Gender plays a crucial role as a determinant of health that influences the prevalence and patterns of chronic diseases among older adults. However, the patterns and prevalence of these diseases may vary across different age groups within the older adult population. Despite the growing burden of chronic diseases among older adults in India, there exists a gap in understanding how these diseases manifest differently based on gender and age. Understanding how chronic diseases affect older adults differently based on gender and age can help identify disparities and inequities in healthcare access, prevention, and treatment. Therefore, the current study makes a comprehensive analysis of the gender and age differentials in the prevalence and pattern of nine chronic diseases among older adults in India. The findings of this study will provide valuable insights into the unique health needs of the aging populations so that policy makers can tailor interventions to promote better health outcomes and quality of life for both men and women in India.

## 2 Methods

### 2.1 Data

This study used data from the first wave of the Longitudinal Ageing Study in India (LASI) (2017‐2018). The LASI is a large‐scale, nationally representative survey that included 72,250 people aged 45 years and over, as well as their spouses (of any age), from all Indian states and union territories (UTs). To identify the final units of observation, the LASI employed multistage stratified sampling. The survey was carried out using a three‐stage sampling design in rural areas and a four‐stage sampling design in urban areas. In the first stage, Primary Sampling Units (PSUs) were selected in ecah state/UT. The PSUs were a Taluk,a Tahesil, or a Community Development Block. In the second stage, villages were selected in the rural areas and wards were selected in the urban areas. In the case of rural areas, the third and final stage involved choosing households from each selected village. In the case of urban areas, the sampling process involved the random selection of one Census Enumeration Block (CEB) from each ward in the third stage. The fourth and final stage involved selecting households from each CEB. LASI provides scientific evidence for chronic health disorders, biomarkers, symptom‐based health conditions, and functional and mental health. The current study is based on 66,606 respondents aged 45 years and older.

### 2.2 Measures

#### 2.2.1 Outcome Variables

##### 2.2.1.1 Nine Self‐Reported Health Conditions

The respondents were asked, “Has any health professional ever diagnosed you with the following diseases?” The diseases were: (1) Hypertension; (2) Diabetes; (3) Cancer; (4) Chronic heart diseases; (5) Stroke; (6) Psychiatric diseases; (7) Chronic lung diseases; (8) Bone and joint diseases; and (9) High cholesterol. The responses were coded as “no” and “yes.”

##### 2.2.1.2 Main Explanatory Variables

The main explanatory variables in this study were age and gender. Age was categorized into three groups: 45–59 years, 60–69 years, and 70 years and older. Gender was categorized as male and female.

#### 2.2.2 Covariates

##### 2.2.2.1 Socioeconomic factors

Education was categorized into four levels: no education, primary, secondary, and graduation and above. Economic status was measured using the monthly per capita expenditure (MPCE) quintile, categorized as: poorest, poor, middle, rich, and richest [21]. Working status was divided into four categories: currently working, currently not working, never worked, and retired. Caste was categorized into three groups: Scheduled caste/Scheduled tribe (SC/ST), Other backward caste (OBC), and Others. Religion was divided into four categories: Hindu, Muslim, Christian, and Other.

##### 2.2.2.2 Lifestyle factors

Alcohol as well as tobacco consumption were both categorized as “no” and “yes.” Similarly, physical inactivity was categorized as “no” and “yes.” Body Mass Index (BMI) was categorized into four groups: underweight, normal weight, overweight, and obese;, and missing response were coded as “missing.” In order to evaluate an individual's engagement with multiple media channels, mass media exposure was added as a variable and categorized as “no” and “yes.”

##### 2.2.2.3 Geographic factors

Geographic characteristics consisted of place of residence and geographic region. Place of residence was classified as urban and rural. Geographic region was categorized into six areas as: north, central, east, northeast, west, and south.

### 2.3 Statistical Analysis

Logistic regression, also known as the logistic or logit model, was used in this study to analyse the relationship between multiple independent variables and a categorical dependent variable. Binary logistic regression is typically used when the dependent variable is dichotomous and the independent variables are either continuous or categorical. If Xi is a set of independent or explanatory variables, and βi's are its coefficients, then the logistic regression equation is given by

$$\mathrm{logit}\;\left(P\right)=\log\;P\;/\;\left(1-P\right)=\beta 0+\beta 1X1+\beta 2X2+\cdots \;\cdots \;\cdots$$

where the probability of an event (having a non‐communicable disease) occurring is P, whereas the probability of the event not occurring is (1‐P).

## 3 Results

Table 1 shows the background characteristics of the study population. Approximately 53% of the sample population was made up of female respondnets, and 46.6% was made up of male respondents. Nearly half (52.1%) of the population was aged 45–59 years. Almost half (47.07%) of the population had no formal education. Only 23.2% had completed secondary and above education. Well over half of the population (73.1 %) followed the Hindu religion. Other backward class (OBC) comprised almost 37.9% of the total population. The population was more or less evenly distributed across the wealth quintiles. More than one‐third (74.4%) of the population was currently married. A majority of the population (64.9%) lived in rural areas. Almost 45% of the study population was currently working, while 28% had never worked. As much as 61.1% of the population was not physically active, whereas 38.9% population was physically active. A majority (82.2%) of the total population consumed alcohol and more than a third (36.4%) had consumed tobacco at some point of time before the survey.

**TABLE 1:** Percentage distribution of self‐reported chronic disease among older adults 45 years and above, LASI, 201‐7‐18 ( n= 66,606).

Background variables	Sample	Percentage (%)
Gender		
Male	31,039	46.60
Female	35,567	53.40
Age (in years)		
45–59	34,704	52.10
60–69	19,211	28.84
70 and above	12,691	19.05
Caste		
ST/SC	22,760	34.17
OBC	25,212	37.85
Others	18,634	27.98
Education		
No formal education	31,353	47.07
Primary education	16,359	24.56
Secondary education	15,465	23.22
Graduation and above	3429	5.15
Place of residence		
Rural	43,240	64.92
Urban	23,366	35.08
MPCE		
Richest	13,239	19.88
Richer	13,412	20.14
Middle	13,371	20.07
Poorer	13,403	20.12
Poorest	13,181	19.79
Marital status		
Currently married	49,555	74.40
Never married	868	1.30
Widowed	14,780	22.19
Others	1403	2.11
Religion		
Hindu	48,711	73.13
Muslim	7806	11.72
Christian	6638	9.97
Others	3451	5.18
Working status		
Currently working	29,997	45.04
Currently not working	14,925	22.41
Never worked	18,634	27.98
Retired	3050	4.58
Mass media exposure		
No	18,736	28.13
Yes	47,870	71.87
Physical inactivity		
No	40,705	61.11
Yes	25,901	38.89
Body mass index		
Underweight	11,002	16.52
Normal weight	31,343	47.06
Overweight	13,133	19.72
Obese	4439	6.66
Missing	6689	10.04
Alcohol Consumption		
No	54,752	82.20
Yes	11,854	17.80
Tobacco Consumption		
No	42,368	63.61
Yes	24,238	36.39

Table 2 depicts the prevalence of hypertension, diabetes, bone and joint diseases, chronic heart diseases, stroke, psychiatric disorders, chronic lung diseases, and high cholesterol among male and female older adults across three age groups (45–59 years, 60–69 years, 70 years, and above).

**TABLE 2:** Prevalence of self‐reported chronic disease among older adults aged 45 years and above by age and gender categories, LASI, 2017‐18 (n=66,606).

		Age categories (in years)		
		45–59	60–69	70 and above
Chronic diseases	Gender	Prevalence in percentage (95% CI)	Prevalence in percentage (95% CI)	Prevalence in percentage (95% CI)
Diabetes	Male	10.3 (8.6,12.2)	14.8 (13.5,16.1)	14.4 (12.9,16.0)
	Female	10.6 (9.1,12.3)	14.3 (12.0,16.9)	13.4 (10.3,17.3)
Hypertension	Male	18.6 (16.7,20.6)	26.7 (25.2,28.3)	29.7 (27.7,31.7)
	Female	25.2 (23.6,26.8)	35.2 (32.9,37.5)	39.9 (36.9,43.0)
Cancer	Male	0.4 (0.2,0.6)	0.6 (0.4,0.9)	0.6 (0.4,0.9)
	Female	0.8 (0.6,1.0)	0.7 (0.5,1.0)	0.9 (0.4,2.1)
Chronic lung disease	Male	5.4 (3.9,7.5)	7.9 (7.0,8.9)	10.6 (9.5,11.8)
	Female	4.4 (3.7,5.1)	6.0 (5.3,6.9)	10.9 (7.9,14.8)
Chronic heart disease	Male	2.5 (2.0,3.1)	5.4 (4.7,6.2)	6.4 (5.2,7.9)
	Female	2.4 (1.9,2.9)	3.8 (3.3,4.4)	5.9 (3.2,10.6)
Stroke	Male	1.7 (1.3,2.4)	3.03 (2.5,3.6)	3.7 (3.1,4.4)
	Female	0.7 (0.5,0.9)	1.6 (1.2,1.9)	3.2 (2.6,4.0)
Bone & joint	Male	9.2 (7.6,11.2)	15.6 (14.4,16.9)	17.1 (15.5,18.9)
	Female	15.6 (14.6,16.6)	21.1 (18.8,23.5)	25.3 (22.1,28.7)
Psychiatric diseases	Male	2.2 (1.7,2.8)	2.6 (2.1,3.1)	2.9 (2.4,3.6)
	Female	2.02 (1.6,2.6)	2.3 (1.9,2.8)	3.7 (3.0,4.6)
High cholesterol	Male	1.9 (1.6,2.1)	2.7 (2.2,3.2)	2.6 (1.6,4.3)
	Female	2.2 (1.9,2.5)	3.0 (2.3,3.9)	1.6 (1.2,2.0)

The prevalence of hypertension, diabetes, bone and joint diseases, chronic heart diseases, stroke, psychiatric disorders, and chronic lung diseases increased with age for both genders. In contrast, the prevalence of high cholesterol varied across age groups without a clear trend.

The prevalence of diabetes was slightly higher among male older adults than their female counterparts in the 70 year 60‐69 years age group (14.8% vs 14.3%). In contrast, in the 45–59 years age group, females (10.6%) had a marginally higher prevalence than males (10.3%).

Female older adults had a higher prevalence of hypertension across all age groups (45–59 years: 25.2% vs. 18.6%, 60–69 years: 35.2% vs. 26.7%, and 70 years and over: 39.9% vs. 29.7%) compared to male older adults. The highest prevalence of hypertension was observed among female participants aged 70 years and above (39.9%). The prevalence of cancer was relatively lower across all age groups. However, gender‐wise, the prevalence was higher among female older adults than male older adults among those aged 45–59 years as well as those aged 70 years and above.

The table shows that the prevalence of chronic lung diseases was higher among male respondents than female respondents among those aged 45–59 years (5.4% vs. 4.4%) and those aged 60–69 years (7.9% vs. 6.0%). In contrast, for adults aged 70 years and above, there was a slightly higher prevalence of chronic lung diseases among female participants (10.9%) than male participants (10.6%).

Chronic heart disease was more prevalent among male respondents than female respondents across all age groups. The prevalence of chronic heart disease and stroke was highest among male respondents aged 70 years and above (6.4% and 3.7%, respectively), compared to female respondents.

The prevalence of bone and joint diseases was higher among female respondents than male respondents across all age groups (45–59 years: 15.6% vs 9.2%; 60–69 years: 21.1% vs 15.6%; and 70 years and above: 25.3% vs 17.1%). Additionally, females aged 70 years and above had a higher prevalence of psychiatric disorders than males (3.7% vs 2.9%). In contrast, male respondents had a slightly higher prevalence of psychiatric disorders than female respondents in the case of those aged 45‐59 years (2.2% vs 2.0%) as well as those aged 60‐69 years (2.6% vs 2.3%).

The prevalence of high cholesterol varied across age groups and genders, with males having higher rates in some age groups and females in others, showing no consistent trend. For example, those aged 45‐59 years (2.2% vs 1.9%) and those aged 60‐69 years (3.0% vs 2.7%), the prevalence of cholesterol was higher among female respondents than male respondents. In contrast, we observed that for those aged 70 years and above, the prevalence of cholesterol was higher among male respondents (2.6% vs 1.6) compared to female respondents.

**FIGURE 1:**
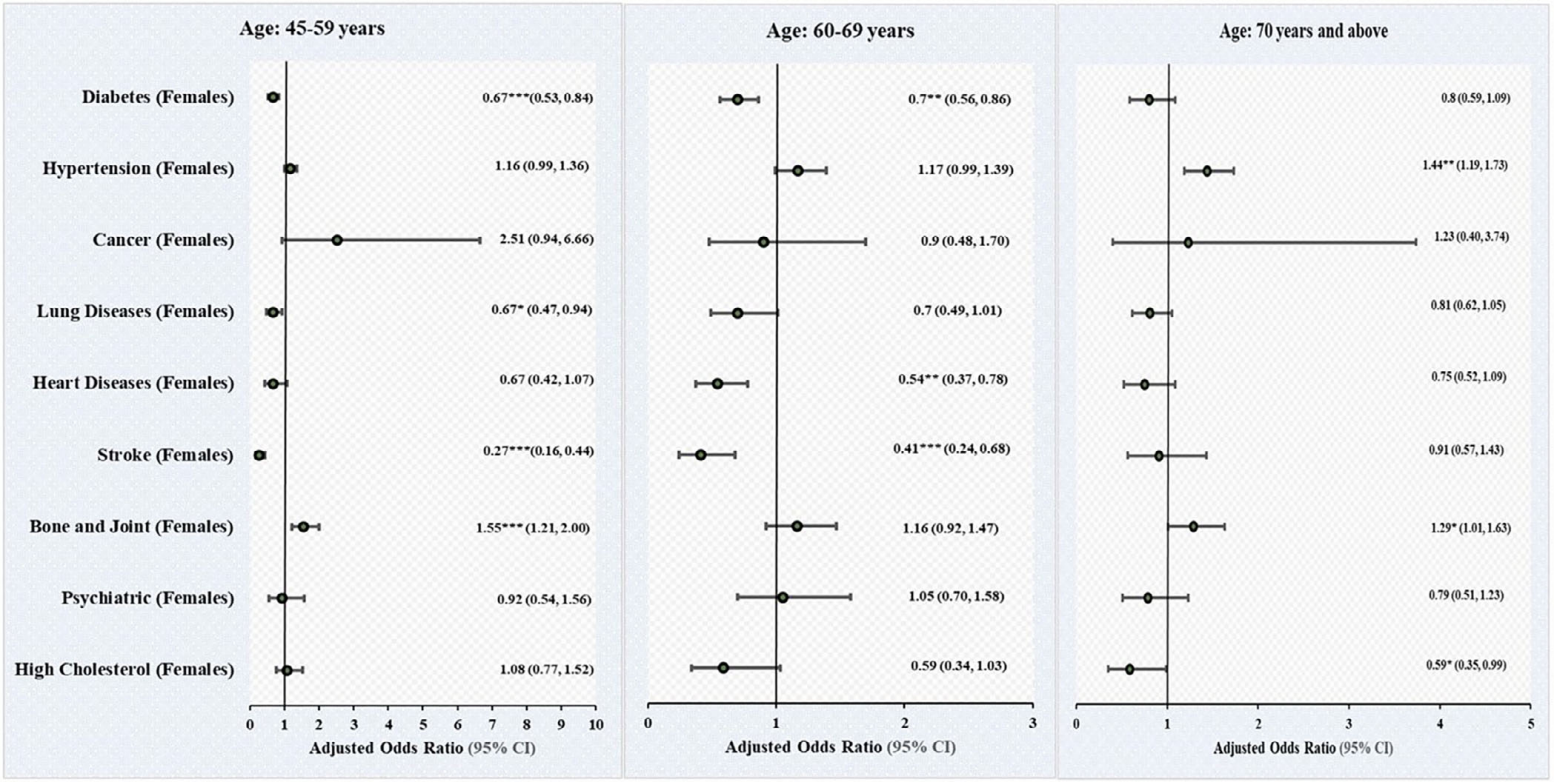
Binary logistic regression estimates for self‐reported chronic diseases by gender and age groups among individuals aged 45 years and above in India (LASI, 2017–18); reference category: Male. ^*^ *p* < 0.05, ^**^ *p* < 0.01; ^***^ *p* < 0.001. AORs (adjusted odds ratios) were adjusted for age, gender, education, marital status, working status, place of residence, caste, MPCE, region, religion, tobacco use, alcohol use, physical activity, mass media exposure, and body mass index.

Table 3 and Figure 1 demonstrate the adjusted binary logistic regression estimates for self‐reported chronic diseases adjusted for various demographic and lifestyle categories. The results show the gender and age‐differential relationship of chronic diseases stratifying older men and women into three age groups (45–59 years, 60–69 years, 70 years and above) after adjusting for various covariates.

**TABLE 3:** Binary logistic regression estimates for self‐reported chronic diseases by gender and age groups among individuals aged 45 years and above in India, LASI, 2017‐18.

		Age categories		
		45–59	60–69	70 and above
Chronic diseases	Gender	AOR (95% CI)	AOR (95% CI)	AOR (95% CI)
Diabetes	Male	Ref.	Ref.	Ref.
	Female	0.67^***^ (0.53,0.84)	0.70^**^ (0.56,0.86)	0.8 (0.59,1.09)
Hypertension	Male	Ref.	Ref.	Ref.
	Female	1.16 (0.99,1.36)	1.17 (0.99,1.39)	1.44^***^ (1.19,1.73)
Cancer	Male	Ref.	Ref.	Ref.
	Female	2.51 (0.94,6.66)	0.9 (0.48,1.70)	1.23 (0.40,3.74)
Chronic lung disease	Male	Ref.	Ref.	Ref.
	Female	0.67^*^ (0.47,0.94)	0.7 (0.49,1.01)	0.81 (0.62,1.05)
Chronic heart disease	Male	Ref.	Ref.	Ref.
	Female	0.67 (0.42,1.07)	0.54^**^ (0.37,0.78)	0.75 (0.52,1.09)
Stroke	Male	Ref.	Ref.	Ref.
	Female	0.27^***^ (0.16,0.44)	0.41^***^ (0.24,0.68)	0.91 (0.57,1.43)
Bone & joint	Male	Ref.	Ref.	Ref.
	Female	1.55^***^ (1.21,2.00)	1.16 (0.92,1.47)	1.29^*^ (1.01,1.63)
Psychiatric diseases	Male	Ref.	Ref.	Ref.
	Female	0.92 (0.54, 1.56)	1.05 (0.70, 1.58)	0.79 (0.51, 1.23)
High cholesterol	Male	Ref.	Ref.	Ref.
	Female	1.08 (0.77, 1.52)	0.59 (0.34, 1.03)	0.59^*^ (0.35, 0.99)

*Note*: AORss(adjusted odds ratios) were adjusted for age, gender, education, marital status, working status, place of residence, caste, MPCE, region, religion, tobacco use, alcohol use, physical inactivity, mass media exposure, and body mass index. Ref: reference category.

* *p* < 0.05.

** *p* < 0.01.

*** *p* < 0.001.

### 3.1 45–59 Years Age Group

In the 45–59 years age group, the odds of diabetes (AOR: 0.67, 95% CI: 0.53–0.84), chronic lung disease (AOR: 0.67, 95% CI: 0.47–0.94), and stroke (AOR: 0.27, 95% CI: 0.16–0.44) were lower among female older adults than male respondents. However, the odds of bone & joint disease (AOR: 1.55, 95% CI: 1.21–2.00) were higher among female older adults than male respondents.

### 3.2 60–69 Years Age Group

In the 60–69 years age group, the odds of diabetes (AOR: 0.70, CI: 0.56–0.86), chronic heart disease (AOR: 0.54, CI: 0.37,0.78), and stroke (AOR: 0.41 CI: 0.24,0.68) were lower among female older adults than male respondents.

### 3.3 70 Years and Above Age Group

In the 70 years and above age group, the odds of hypertension (AOR: 1.44, CI: 1.19–1.73) and bone and joint diseases (AOR: 1.29, CI: 1.01–1.63) were higher among female older adults than male respondents. In contrast, the odds of high cholesterol (AOR: 0.59, CI: 0.35,0.99) were lower among female older adults than male respondents.

## 4 Discussion

The current study revealed significant gender and age‐related differences in the prevalence and odds of chronic diseases like diabetes, hypertension, cancer, chronic lung diseases, chronic heart diseases, bone and joint diseases, psychiatric disorders, and high cholesterol among older adults. Older males had a higher prevalence of diabetes, chronic heart disease, and stroke, particularly in the 60–69 years and 70+ years age groups, indicating a need for interventions in this population. In contrast, older females, especially those aged 70 years and above, were more prone to hypertension, bone and joint diseases, and certain cancers. These differences underscore the necessity for gender‐specific healthcare strategies.

The study findings show that female older adults had a higher prevalence of hypertension across all age groups compared to male older adults. This is in contrast to the National Health and Nutrition Examination Survey (NHANES) data from 2017 to 2020, according to which, a higher percentage of males than females had hypertension up to the age of 64 years, whereas a higher percentage of females than males had hypertension among those aged 65 years and older [19]. Interestingly, we observed the prevalence of hypertension being the highest among female participants aged 70 years and above (39.9%). Additionally, in that age group, the odds of hypertension were also higher among female respondents than male respondents. A previous study observed that females experience a more rapid progression in blood pressure measurements over their lifetime, which may suggest a gender‐specific genetic predisposition to hypertension [22, 23]. One prior study demonstrated that gender‐specific genetic risk factors were more strongly linked to hypertension risk in women than in men, particularly for early‐onset hypertension. Specifically, a lower genetic burden provides greater protection against hypertension in women compared to men [23]. Furthermore, the effects of elevated blood pressure appear to be gender‐specific, with emerging evidence suggesting that hypertension represents a more significant cardiovascular risk factor in females [24].

The prevalence of diabetes was slightly higher among male older adults than their female counterparts aged 70 years and above as well as those aged 60–69 years. In contrast, in the 45–59 years age group, females had a marginally higher prevalence than males. A previous study demonstrated that the prevalence of diabetes increased with age and was higher in males compared to females [25]. Another prior study observed that diabetes was highly prevalent in those aged 65–74 years [26]. Our results revealed that the odds of diabetes were lower among female older adults than male respondents among those aged 45–59 years and those aged 60–69 years. Diabetes accounts for a greater population‐attributable risk among American women than men, with estimates of 21% and 14%, respectively [27]. The reduction in life expectancy associated with diabetes decreases as the age at diagnosis increases. Nonetheless, due to the growing prevalence of diabetes, the total number of years lived with the condition has risen significantly, with an increase of 156% for men and 70% for women overall [20].

Our results further show that for those aged 45–59 years and aged 60–69 years, the prevalence of high cholesterol was higher among female respondents than male respondents. The existing literature suggests that while women generally have higher total cholesterol (TC) levels than men, they tend to have lower triglycerides (TGs) and a lower TC‐to‐HDL‐C ratio, along with higher HDL‐C levels on average [28, 29, 30, 31, 32, 33]. Interestingly, we observed that for those aged 70 years and above, the prevalence of high cholesterol was higher among male respondents compared to female respondents. Additionally, our findings show that in those aged 70 and above, the odds of high cholesterol were lower among female older adults than male respondents. One previous study revealed that for those aged 60 years and over, the prevalence of high total cholesterol was lower among men than women [34]. However, the study reported that in men, the prevalence of high total cholesterol was the highest among those aged 40–59 years, compared to those aged 60 years and older. Among women, the prevalence was elevated in both the 40–59 years age group and 60 years and older age group compared to the 20–39 years age group [34]. Beyond gender differences, lipid levels also fluctuate with age. Total cholesterol generally increases with age, with a potential threshold occurring around 50–59 years [35]. However, when comparing younger adults (aged 18–29 years) with older adults (aged 70 years and above), the latter tend to have higher levels of both TGs and HDL‐C [36].

Our findings present the evidence that the prevalence of bone and joint diseases is higher among female respondents across all age groups than male respondents. Concordantly, a previous study reported that gender, a significant systemic factor, is often considered a risk factor for osteoarthritis due to its higher prevalence in women compared to men [37]. We observed that the highest prevalence of bone and joint diseases was among female participants aged 70 years and above. A previous study demonstrated that rheumatoid arthritis disproportionately impacted women, who had a 2–3‐fold higher risk compared to men. The highest prevalence of RA was observed in women over the age of 65 years [38, 39]. Interestingly, our results revealed that the odds of bone and joint disease were significantly higher among female older adults than male respondents in those aged 45–59 years, and those aged 70 years and older. A previous study suggests that women are more likely to have arthritis compared to men [40]. Another research study demonstrated that rheumatoid arthritis, a severely debilitating chronic inflammatory immune disorder, primarily affects women and the elderly [41]. In the NHANES III survey, where hip bone mineral density was measured, men exhibited higher average bone mineral density across all racial groups compared to women. As a result, the prevalence of low bone mass was higher in women than in men and more pronounced in non‐Hispanic Whites compared to blacks [42, 43]. Age‐specific incidence rates for hip fractures are notably higher in women than in men up to the ninth decade of life [44].

Our results show that chronic heart disease was more prevalent among male respondents than female respondents across all age groups. Recent studies have demonstrated that the age‐adjusted prevalence of heart disease and stroke are higher in males compared to females [19, 45]. Conversely, some previous studies indicated that the prevalence of angina was higher among female respondents compared to male respondents [46, 47]. Our study revealed that the odds of chronic heart disease were lower among female older adults than male respondents among those aged 60–69 years, whereas the odds of stroke were lower among female older adults than male respondents among those aged 45–59 years and those aged 60–69 years. A previous study demonstrated that females had lower odds of heart disease compared to males, while they had higher odds of experiencing angina [46]. We also found that the prevalence of chronic heart disease and stroke was the highest among male respondents aged 70 years and above. One prior study revealed that cardiovascular disease typically becomes clinically evident in middle age or later. At age 40, the lifetime risk of developing incident CVD is approximately 66% for men and slightly above 50% for women [48]. Estimates obtained from the Framingham Study cohort suggested that the lifetime risk of stroke is approximately 20% for women and 17% for men who reach age 55 without a prior stroke, indicating that one in five women and one in six men will experience a stroke during their remaining lifetime [49, 50].

In our study, the prevalence of chronic lung diseases was higher among male respondents than female respondents among those aged 45–59 years and those aged 60–69 years. Additionally, among those aged 45‐49 years, the odds of chronic lung disease were also higher among male respondents compared to female older adults. In line with our findings, a previous study demonstrated that the overall prevalence of chronic lung diseases was higher among male respondents compared to female respondents [47]. Another study demonstrated that the age‐specific prevalence of COPD increased sharply after age 30, with a more pronounced rise in men, reaching its peak among men aged 80 years and older [51]. The study also highlighted that asthma prevalence was the highest in the 75–79 years age group at 12.1% among men and 10.4% among women [51]. In contrast, our findings show that, among older adults aged 70 years and above, there was a slightly higher prevalence of chronic lung diseases among female than male participants.

The study findings on the prevalence and odds of chronic diseases among older adults reveal significant gender and age‐specific disparities that have important clinical implications. Understanding these patterns is crucial for tailoring healthcare strategies, improving patient outcomes, and optimizing resource allocation in clinical practice.

## 5 Conclusion

The current study findings underscore the significant gender and age‐related disparities in the prevalence and odds of various chronic conditions among older adults. The findings underscore the complexity of managing chronic conditions in older adults, particularly given the gender and age differences. Public health initiatives, clinical practices, and research efforts must be tailored to address these disparities. Recognizing differences in disease prevalence and patterns between genders can aid in the early detection and management of chronic disease. By focusing on the unique needs of different demographic groups, particularly in terms of gender and age, healthcare systems can improve outcomes for older adults through reducing the burden of chronic diseases and enhancing the overall quality of life. These insights also point to the need for ongoing research to better understand the underlying factors driving these disparities and to develop more effective, targeted interventions. The LASI dataset is a rich source of information for understanding health and socioeconomic conditions in India. Nevertheless, there were certain limitations inherent in the study design. The results that were used for estimating disease prevalence were derived from self‐reported responses to a structured questionnaire, which may have introduced biases related to recall and social desirability or may have been subject to misreporting. Another constraint was that a considerable proportion of respondents, particularly those from socioeconomically disadvantaged backgrounds, had limited literacy and unstable employment. Such factors may have affected the respondents’ awareness and comprehension of health‐related questions, potentially influencing response accuracy. Nonetheless, the large‐scale nature of LASI mitigates these limitations to some extent by ensuring broad representativeness. Future research should consider integrating supplementary validation methods, such as biomarker data or qualitative assessments, to enhance the reliability of self‐reported information.

## Author Contributions

MB, LKD and PD conceptualized the study; MB, PM, LKD and PD were responsible for writing the original draft; PM, LKD, WA, SJ and PD were involved with formal analysis of the data. MB, LKD, PM, SD, WA, SJ and PD were responsible for editing the final draft. All the authors have read and approved the manuscript.

## Ethics Statement

This analysis is based on a secondary dataset with no identifiable information on the survey participants. This dataset is available in the public domain for research use; hence, no approval was required from any institutional review board, as there is no question of human subject protection arising in this case.

## Conflicts of Interest

The authors declared that they have no competing interests.

## Data Availability

The data utilized in this study is publicly available and can be accessed online upon submission of a formal request from https://www.iipsindia.ac.in/content/LASI‐data. The International Institute for Population Sciences (IIPS), Mumbai, served as the nodal agency for LASI Wave 1.
